# Functionalization of Calcium Sulfate/Bioglass Scaffolds with Zinc Oxide Whisker

**DOI:** 10.3390/molecules21030378

**Published:** 2016-03-18

**Authors:** Cijun Shuai, Jianhua Zhou, Dan Gao, Chengde Gao, Pei Feng, Shuping Peng

**Affiliations:** 1 State Key Laboratory of High Performance Complex Manufacturing, Central South University, Changsha 410083, China; shuai@csu.edu.cn (C.S.); zhouyx@csu.edu.cn (J.Z.); gaochengde@csu.edu.cn (C.G.); fengpei@csu.edu.cn (P.F.); 2 Shenzhen Research Institute, Central South University, Shenzhen 518057, China; 3 School of Basic Medical Science, Central South University, Changsha 410078, China; gd146611004@csu.edu.cn

**Keywords:** zinc oxide whisker, antibacterial activity, mechanical property, biocompatibility, scaffolds

## Abstract

There are urgent demands for satisfactory antibacterial activity and mechanical properties of bone scaffolds. In this study, zinc oxide whisker (ZnOw) was introduced into calcium sulfate/bioglass scaffolds. Antimicrobial behavior was analyzed using *Escherichia coli (E. coli)* and *Staphylococcus aureus* (*S. aureus*). The results showed that the scaffolds presented a strong antibacterial activity after introducing ZnOw, due to the antibacterial factors released from the degradation of ZnO. Moreover, ZnOw was also found to have a distinct reinforcing effect on mechanical properties. This was ascribed to whisker pull-out, crack bridging, crack deflection, crack branching and other toughening mechanisms. In addition, the cell culture experiments showed that the scaffolds with ZnOw had a good biocompatibility.

## 1. Introduction

The lack of antibacterial activity easily leads to bacterial infection in bone scaffolds [1,2,3]. In recent years, the bacterial infections gradually increased in bone transplantation, leading to the failure of treatment [4,5,6]. Therefore, antibacterial activity has resulted in an urgent need for bone scaffolds. ZnO is a novel type of antibacterial agent with strong antibacterial activity, long life expectancy, high safety and low cost [7,8,9,10]. It can be employed for developing antibacterial tissue repair materials [11]. ZnO can react chemically with water and generate antibacterial factors during the process of degradation. These antibacterial factors are able to provide the antibacterial effect by disrupting bacterial membranes [12,13,14].

Zn is a multifunctional mineral element which is essential to the completion of bone regeneration and many important biological activities [15]. Moreover, ZnO whisker (ZnOw) possesses high strength, modulus and no crystal defects, which has attracted lots of interests as a reinforcing phase in bone scaffolds [16]. Calcium sulfate (CaSO_4_) has been widely used to bone defect regeneration due to the good osteoconductivity, complete degradation and no stimulation for body [17,18]. Its bone bonding ability and stability can be improved by introducing bioglass [19,20,21].

In recent years, ZnOw as an antibacterial agent or reinforcing phase has been studied. Fang *et al.* reported that ZnOw was incorporated in the dental materials and revealed the high antibacterial effect against streptococcus mutans [22]. Jin *et al.* added ZnOw into hydroxyapatite/tricalcium phosphate biphasic bioceramic, it was shown that mechanical strength and densification were greatly enhanced in the composite [23]. Bini *et al.* studied the effect of the ZnO on biological performance of 58S bioglass and verified that its bioactivity and biocompatibility were significantly improved [24].

In the study, needle-like ZnOw was incorporated into CaSO_4_/bioglass scaffolds and expected to enhance simultaneously its antibacterial activity and mechanical properties. The phase composition and micromorphology of scaffolds with different contents of ZnOw were detected through X-ray diffraction and scanning electron microscopy. The influence of ZnOw on antibacterial activity was investigated using *E. coli* and *S. aureus*. The strength and toughness of the scaffolds were measured by pressure test and indentation method, respectively. In addition, the cell culture was performed to evaluate adhesion and growth behaviors of osteoblast-like cells on the scaffolds.

## 2. Results and Discussion

### 2.1. Scaffolds Fabrication

**Figure 1:**
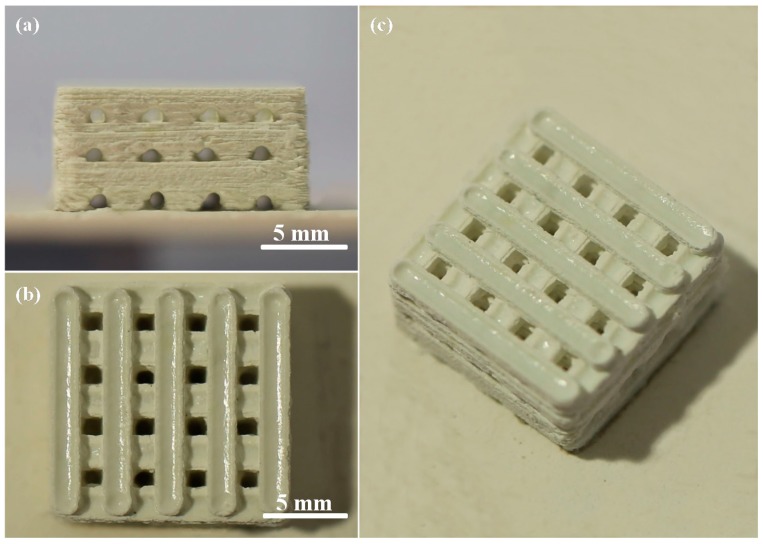
(**a**) Front view; (**b**) top view and (**c**) oblique view of the CaSO_4_/bioglass scaffold with zinc oxide whisker (ZnOw).

Porous CaSO_4_/bioglass scaffolds with ZnOw were manufactured using selective laser sintering (SLS) (Figure 1). The size of cubic scaffolds was about 14 mm × 14 mm × 6.5 mm (length × width × height). The pore channel was completely interconnected and formed 3D porosity by branching orthogonally. The wall thickness and pore size were about 1.8 mm and 1.2 mm, respectively. It had been reported that the pore size above 300 μm is essential for new bone ingrowth and vascularization of constructs [25].

### 2.2. Antibacterial Activity

**Figure 2:**
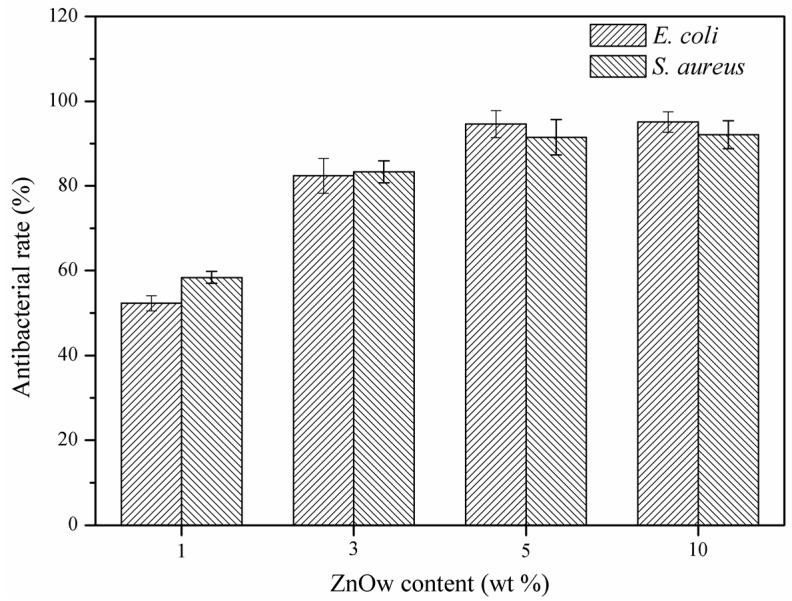
Antibacterial rate for the scaffolds with different contents of ZnOw.

The antibacterial rate of scaffolds with different contents of ZnOw was investigated using *Escherichia coli (E. coli)* and *Staphylococcus aureus* (*S. aureus*) (Figure 2). The results indicated that ZnOw could provide the scaffolds with remarkable antibacterial activity and gradually enhanced with the ZnOw content increasing. The antibacterial rate arrived to a peak value in 5 wt % ZnOw group and there was no apparent change for the antibacterial rates between the 5 wt % and 10 wt % ZnOw groups. The scaffolds showed a distinct antibacterial effect against *E. coli* and *S. aureus* only after introducing ZnOw, indicating that it played key roles in the improvement of antibacterial property. It could be ascribed to that antibacterial factors were released from the degradation of ZnO. These antibacterial factors might react with the bacterial envelope components or the free ions in the bacteria, which could restrain the bacteria growth or even kill them [26]. Moreover, the Zn ions, which had certain of antibacterial activity similar to that of Ag ions, might be conducive to the antibacterial effect of ZnO [27].

### 2.3. Mechanical Properties

**Figure 3:**
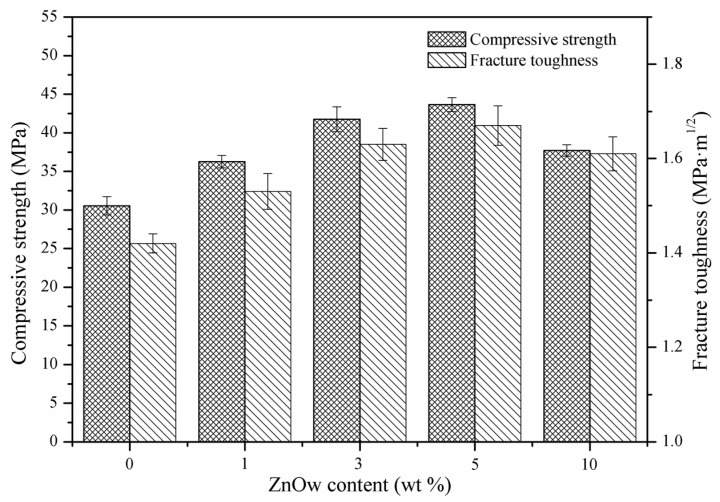
Compressive strength and fracture toughness of the scaffolds with different contents of ZnOw.

The compression strength and fracture toughness indicated that ZnOw had a significant effect on mechanical properties of scaffolds (Figure 3). In the 1 wt % and 3 wt % ZnOw groups, the compression strength and fracture toughness were clearly higher than those in the group without ZnOw. In the 5 wt % ZnOw group, both two kinds of mechanical properties were higher than those of the other groups, which were 43.66 ± 0.91 MPa and 1.67 ± 0.04 MPa·m^1/2^, respectively. While the properties of scaffolds appeared decline with further increasing of ZnOw to 10 wt %.

### 2.4. Composition and Microstructure

**Figure 4:**
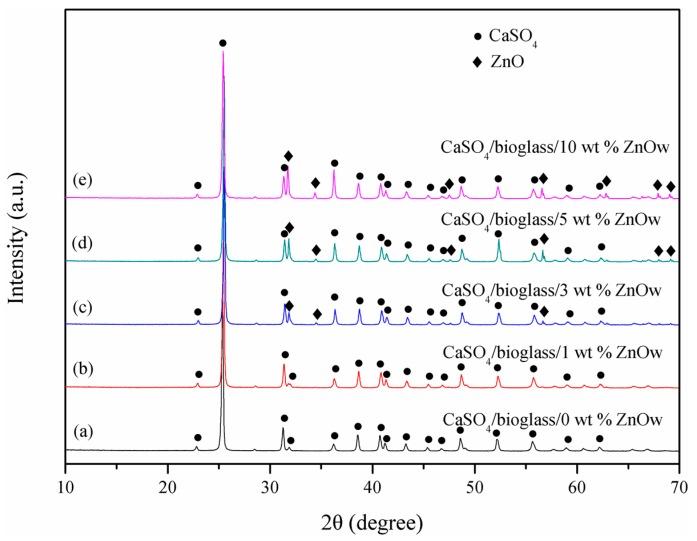
XRD spectra for the scaffolds with (a) 0 wt %; (b) 1 wt %; (c) 3 wt %; (d) 5 wt % and (e) 10 wt % ZnOw.

Phase components of the scaffolds with different contents of ZnOw were determinated and plotted in XRD spectra (Figure 4). CaSO_4_ phase, as a predominant component of the scaffolds, was clearly recorded in all the spectra. Moreover, the characteristic peaks of ZnO were visible in all the scaffolds with ZnOw, except in 1 wt % ZnOw group (this content probably below detectable limit). In addition, no other phase was observed in the scaffolds. It was indicated that ZnO were chemically compatible with CaSO_4_ during sintering which did not occur chemical reaction.

**Figure 5:**
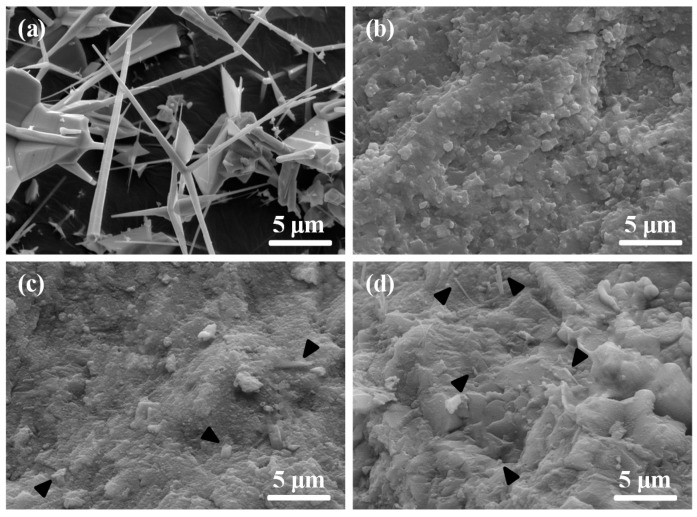

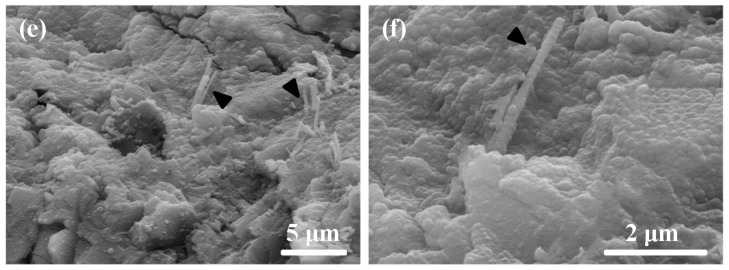
Morphology of (**a**) the initial ZnOw powder and typical fracture surfaces of the scaffolds with (**b**) 0 wt %; (**c**) 3 wt %; (**d**) 5 wt %; (**e**) 10 wt % ZnOw and (**f**) matrix remnants on the ZnOw. The black triangles indicate the ZnOw.

The initial ZnOw (Figure 5a) had a specific needle-like structure and its diameter and length were ~0.5 μm and ~10 μm, respectively. The length/diameter ratio of whiskers was in the scope of 10–100. ZnOw still kept needle-like structure as expected after sintering. Moreover, it could uniformly dispersed in the scaffolds containing up to 5 wt % of ZnOw (Figure 5c,d). The whiskers were embedded into the matrix and only litter tip was observed. The whiskers agglomerated in the scaffolds with 10 wt % ZnOw (Figure 5e). The matrix remnant on the pull-out whiskers indicated that there were the strong bonding interfaces between the matrix and ZnOw (Figure 5f). The improvement of compressive strength could be attributed to the corresponding stress transfer by ZnOw. The high stress region could be released when compressive stress of this region was transferred to other region, and thus that the scaffolds possessed an enhancing compressive resistance. While excessive ZnOw would cause the agglomeration and lower the effective interfacial bonding between the matrix and ZnOw, resulting in some inner defects such as cracks and voids. This would cause stress concentration, resulting in mechanical strength declining.

**Figure 6:**
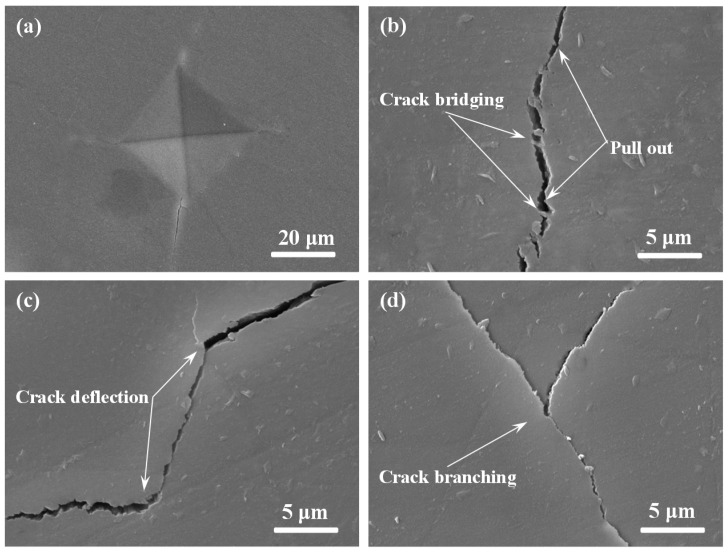
Toughening mechanisms observed on the polished scaffolds with 5 wt % ZnOw: (**a**) Vickers indentation; (**b**) crack bridging and pull-out; (**c**) crack deflection; and (**d**) crack branching.

To provide a detailed understanding about the enhanced fracture toughness in the scaffolds with ZnOw, the typical crack propagation path was caused using the Vickers indentation to study the effect of ZnOw on the crack propagation model (Figure 6). It could be observed that various toughening mechanisms appeared on the scaffolds such as whiskers pull-out, crack bridging, crack deflection and crack branching (Figure 6b–d). These toughening mechanisms could absorb plentiful fracture energy and hinder further propagation of the crack in the matrix, which played an important role to enhance fracture toughness.

### 2.5. Biocompatibility

**Figure 7:**
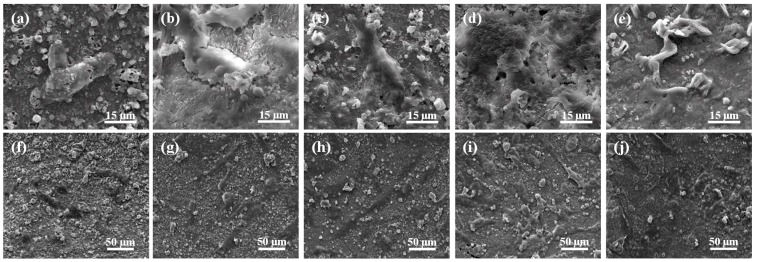
Morphology of osteoblast-like cells seeded on the scaffolds with (**a**,**f**) 0 wt %; (**b**,**g**) 1 wt %; (**c**,**h**) 3 wt %; (**d**,**i**) 5 wt %; and (**e**,**j**) 10 wt % ZnOw for one day.

**Figure 8:**
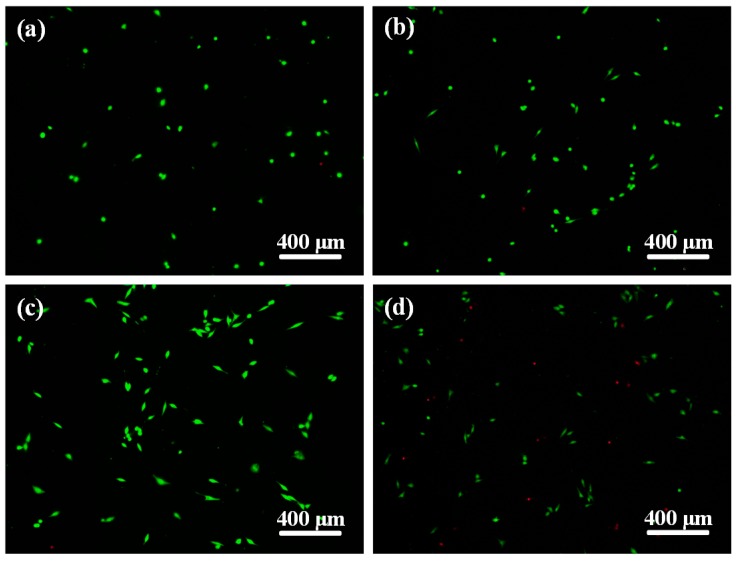
Fluorescent images of cells cultured on the scaffolds with: (**a**) 0 wt % (control); (**b**) 3 wt %; (**c**) 5 wt % and (**d**) 10 wt % ZnOw for one day.

Cell adhesion behavior on the scaffolds with different contents of ZnOw was studied using human osteoblast-like cells after incubation of one day and shown in Figure 7. The results indicated that the cells on scaffolds with ZnOw presented a good cell adhesion and cytoplasmic extension compared with that on scaffolds without ZnOw. As the ZnOw increasing from 1 wt % to 5 wt % (Figure 7b–d,g–i), the cells appeared obvious lamellipodia extensions with cell flattening and gradually spread out on the whole surface, demonstrating that the adhered cells closely contacted with the scaffolds. A sharp decrease for the cell expansion degree was observed when ZnOw were further increased to 10 wt % (Figure 7e,j). Cell morphology starts to get affected adversely at this content of ZnOw. The Significant cytoplasmic extension on the scaffolds with ZnOw was further demonstrated in the Live/Dead stained fluorescent image (Figure 8). Besides, the majority of cells remained live (green staining) after their incubation on the scaffolds when ZnOw content were not exceeding 5 wt %, only very few dead cells (red staining) were detectable (Figure 8b,c). The number of dead cells in 10 wt % ZnOw group was clearly higher compared to the other groups (Figure 8d). It was indicated that 5 wt % ZnOw in the scaffolds had an optimum promotion effect for cell attachment and growth, and presented non-toxicity to the cells. It was indicated that 5 wt % ZnOw in the scaffolds had an optimum promotion effect for the cell attachment and growth.

The improvement of cell viability could be attributed to the release of Zn ions from ZnOw during degradation. Zn was an essential trace element which had a stimulatory effect for multiple cell activities *in vitro* and *in vivo* such as cell attachment and proliferation. It should be taken into consideration that ZnO at elevated concentrations would be some cytotoxic effects for cell growth.

### 2.6. Bioactivity Evaluation

**Figure 9:**
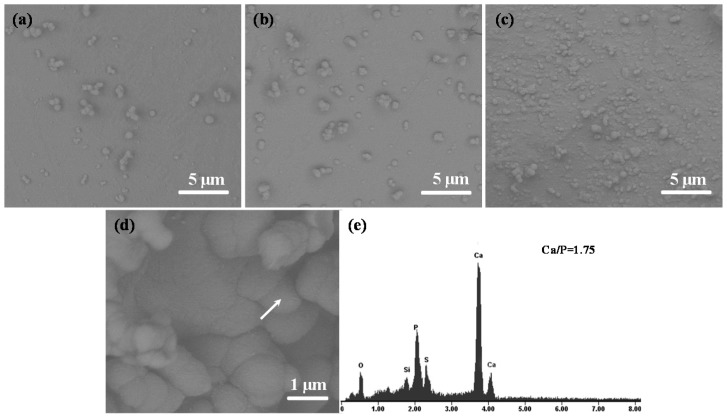
Morphology of the scaffolds with 5 wt % ZnOw after soaking in simulated body fluid (SBF) for (**a**) one day; (**b**) three days and (**c**,**d**) five days; (**e**) energy dispersive spectroscopy (EDS) analysis of precipitates on the scaffolds. The arrow indicates the precipitates for EDS analysis.

The prerequisite of bone scaffolds bonding to living bone is the formation of bone-like apatite on its surface in the body, which was a primary indicator of bioactivity [28]. Hence, the measurement for formation ability of apatite is an important step to evaluate bioactivity of the scaffolds. Morphologies of the scaffolds with ZnOw after immersion for different periods in SBF were shown in Figure 9. The globular precipitates with a typical feature of bone-like apatite homogenously were distributed on the scaffolds. The precipitates gradually increased to cover the entire surface with the extension of immersion periods (Figure 9a–c). Energy dispersive spectroscopy (EDS) measurement was carried out in order to analyze and determine the composition of the precipitated layer. The results from the EDS analysis confirmed the forming of apatite layer on the scaffolds after soaking in SBF due to Ca/P ratio of precipitates being close to that of apatite (1.67) (Figure 9d,e).

## 3. Experimental Procedures

### 3.1. Materials and Preparation

CaSO_4_ powder (purity: 99%) was purchased from Alfa Aesar Co., Ltd. (Shanghai, China). Bioglass powder (prepared by the sol-gel processes) was provided by KunShan Chinese New Material Technology Co. Ltd. (KunShan, China), which had the chemical compositions of 63% SiO_2_, 9% P_2_O_5_ and 28% CaO in molar percentages. ZnOw (purity: >99%) was obtained from Hefei Aijia New Materials Technology Co. Ltd. (Hefei, China), which could be prepared using metallurgical and chemical methods at present [29]. Its length and diameter is approximately 10 μm and 0.5 μm, respectively.

In the mixing process of composite powders, 95 wt % CaSO_4_ and 5 wt % bioglass were first dispersed uniformly by ball-milling for three hours [30]. Then, the ZnOw (1, 3, 5, and 10 wt %) were respectively added to the milled CaSO_4_/bioglass powders. The mixed powders (CaSO_4_/bioglass/ZnOw) were dispersed in anhydrous ethanol for three hours by ultrasonication. Subsequently, the powders in suspension liquid were filtered and dried at 60 °C for 5 h in an oven (DZ-3, Taisite Instrument Co., Ltd., Tianjin, China).

A self-developed SLS system was applied to fabricate 3D scaffolds. The system was composed of a three-dimensional motion platform, a focus system and a control system. The maximum output power of laser was 100 W. The minimum spot diameter could reach 100 μm through the laser focus system. In the sintering process, a CO_2_ laser beam was carried out in a selected area of the powder bed. The motion platform lowered about 100 μm after a slice of powder being sintered each time, and then the new powder was spread out on the sintered layer. The scaffolds with interconnected porous structure were built up by repeating above steps and removing non-sintered powder. The laser sintering parameters were expressed in Table 1.

### 3.2. Antibacterial Behavior

Antibacterial activity of scaffolds was evaluated by the bacterial culture test. The scaffolds with different contents of ZnOw (1, 3, 5, and 10 wt %) were used as four experimental groups. The scaffolds without ZnOw were used as a blank control. Eight scaffolds were prepared for each group. All scaffolds were polished using the abrasive paper and polishing machine. Before the experiment, the scaffolds were sterilized in the steam autoclave and dried on the superclean bench.

The 10 µL solution with *E. coli* (ATCC, Rockville, MD, USA) and 10 µL solution with *S. aureus* (ATCC) at a density of 1 × 10^7^ CFU/mL was placed onto scaffolds of any groups, respectively. Then, the sterilized polyethylene film was applied to cover the scaffolds surface. The bacteria on the scaffolds were collected after anaerobic cultivation at 37 °C for 24 h. The number of viable bacteria on scaffolds was measured by Colony Forming Unit (CFU) plate count method. The antibacterial rate could be calculated by Equation (1).

r% = [(b − c)/b] × 100%(1)

where r% is on behalf of the antibacterial rate and b is on behalf of the bacterial number of the blank control group and c is on behalf of the bacterial number of the experimental group.

### 3.3. Characterization

The phase composition of scaffolds was characterized through applying X-ray diffraction technique (XRD, Bruker AXS, Karlsruhe, Germany) with CuKα radiation at a scanning speed of 8°/min, a scan step of 0.01°, a current of 250 mA and an accelerating voltage of 40 kV. The XRD spectra were performed in the 2θ range between 10° and 70°. The microstructure of the scaffolds was observed through a scanning electron microscope (SEM, TESCAN MIRA3, Brno, Czech Republic), which could also be used for elemental analysis by equipping EDS. The apparatus was operated at current of 40 mA and accelerating voltage of 20 kV. Because of weak conductivity of the scaffolds, the surface of each sample was coated with a thin layer of platinum before testing.

Compressive strength testing of the scaffolds was performed using an universal testing apparatus (WD-01, Zhuoji Instruments, Shanghai, China) at a rate of 0.5 mm·min^−1^ and a maximum loading of 100 N. The fracture toughness was determined on smoothly polished surfaces through the indentation method (HXD-1000TM, Shanghai Taiming Optical Instrument, Shanghai, China). For toughness measurement, the crack length from the indentation center was obtained when indentation tests were performed with indentation loading of 4.9 N. Based on the measured radial crack length, the fracture toughness (*K_IC_*) was assessed following the established Equation (2) proposed by Evans and Charles [31].

*K_IC_* = 0.0824(*P*·*c*^−3/2^) (in MPa·m^1/2^) (2)

where *P* is on behalf of the indentation loading (N) and c is on behalf of the radial crack length (m).

### 3.4. Cell Culture

Human osteoblast-like cell lines (MG63, ATCC) were used for this test. Prior to seeding on the scaffolds, the cells were revived by cultivating in Dulbecco’s modified Eagle’s medium which was added with 100 μg/mL Streptomycin, 100 U/mL penicillin and 10% fetal bovine serum. The cell line was incubated in CO_2_ incubator (Thermo, Waltham, MA, USA) for further proliferation and growth at 5% CO_2_, 37 °C and 90% humidity. The medium was replaced every two days. The cells in confluent monolayer were detached from the culture plates using 0.5% trypsin and 0.2% trypsin-ethylenediaminetetraacetic acid (EDTA) solution.

All the scaffolds were sterilized at 121 °C and 103 kPa pressure for 30 min in steam autoclave. The sterilized scaffolds were placed in culture plates, and then the cells at a density of 5 × 10^3^ cells/cm^2^ were seeded on the wetted scaffolds. Subsequently, the scaffolds with cells were cultivated in a CO_2_ incubator with suitable cells culture conditions. One day later, the scaffolds were removed out from culture plates and cleaned twice with phosphate buffer saline (PBS), and then placed in PBS solution containing 1.5% glutaraldehyde. The cells adhered on the scaffolds were dehydrated using a sequence of alcohol solution for 15 min twice and further dried using Hexamethyldisilazane for 15 min. The dried scaffolds were sputter-coated with platinum and examined through SEM.

Cell viability tests of the scaffolds was measured by using alive/dead cells assay kit. The scaffolds with MG63 cells at a density of 5 × 10^3^ cells/cm^2^ were cultured at 37 °C and 5% CO_2_ for one day. Ethidium homodimer (EthD-1) and calcein AM were mixed with PBS, added to the scaffolds with cultured cells, and incubated for 30 min at 37 °C in 5% CO_2_. Afterwards, the scaffolds were washed with PBS and observed using an epifluorescence microscope (Olympus, Tokyo, Japan). EthD-1 (red) and Calcein AM (green) respectively represented dead and live cells.

### 3.5. Bioactivity

The bioactivity of scaffolds was evaluated in simulated body fluid (SBF) which was an inorganic aqueous solution with corresponding ions concentration close to those of blood plasma [32]. The scaffolds were kept in SBF at pH 7.4 and 37 °C (±0.1 °C) for one, four and seven days, respectively. After soaking, the scaffolds were cleaned using distilled water and then dried at 50 °C in an oven. The surface morphology changes and the precipitation of apatite on the scaffolds were detected for each period of time by SEM and EDS microanalysis.

## 4. Conclusions

The ZnOw was used to improve CaSO_4_/bioglass scaffolds which were prepared via SLS. Significant antibacterial activity was confirmed when the scaffolds with ZnOw were tested against *E. coli* and *S. aureus*. Moreover, the ZnOw obviously enhanced compression strength and fracture toughness. The improvement of mechanical properties could be ascribed to the combination of multiple toughening mechanisms including whiskers pull-out, crack bridging, crack deflection and crack branching. In addition, the MG63 cells culture revealed that the ZnOw could enhance cells attachment, extension and interconnection behavior. The bioactivity tests showed a good forming ability of apatite in the scaffolds with ZnOw.

## Figures and Tables

**Table 1:** Laser sintering parameters.

Spot Diameter (mm)	Laser Power (W)	Scan Speed (mm·min^−1^)	Scan Spacing (mm)	Spot Diameter (mm)
100	7.0	0.1	3.0	0.8
